# Self-Sampling for Human Papillomavirus Testing among Non-Attenders Increases Attendance to the Norwegian Cervical Cancer Screening Programme

**DOI:** 10.1371/journal.pone.0151978

**Published:** 2016-04-13

**Authors:** Espen Enerly, Jesper Bonde, Kristina Schee, Helle Pedersen, Stefan Lönnberg, Mari Nygård

**Affiliations:** 1 Department of Research, Cancer Registry of Norway, Oslo University Hospital, Oslo, Norway; 2 Department of Pathology, Copenhagen University Hospital Hvidovre, Hvidovre, Denmark; 3 Clinical Research Centre, Copenhagen University Hospital Hvidovre, Hvidovre, Denmark; 4 Cervical Cancer Screening Programme, Cancer Registry of Norway, Oslo University Hospital, Oslo, Norway; Rudjer Boskovic Institute, CROATIA

## Abstract

Increasing attendance to screening offers the best potential for improving the effectiveness of well-established cervical cancer screening programs. Self-sampling at home for human papillomavirus (HPV) testing as an alternative to a clinical sampling can be a useful policy to increase attendance. To determine whether self-sampling improves screening attendance for women who do not regularly attend the Norwegian Cervical Cancer Screening Programme (NCCSP), 800 women aged 25–69 years in the Oslo area who were due to receive a 2^nd^ reminder to attend regular screening were randomly selected and invited to be part of the intervention group. Women in this group received one of two self-sampling devices, Evalyn Brush or Delphi Screener. To attend screening, women in the intervention group had the option of using the self-sampling device (self-sampling subgroup) or visiting their physician for a cervical smear. Self-sampled specimens were split and analyzed for the presence of high-risk (hr) HPV by the CLART® HPV2 test and the *digene®* Hybrid Capture (HC)2 test. The control group consisted of 2593 women who received a 2^nd^ reminder letter according to the current guidelines of the NCCSP. The attendance rates were 33.4% in the intervention group and 23.2% in the control group, with similar attendance rates for both self-sampling devices. Women in the self-sampling subgroup responded favorably to both self-sampling devices and cited not remembering receiving a call for screening as the most dominant reason for previous non-attendance. Thirty-two of 34 (94.1%) hrHPV-positive women in the self-sampling subgroup attended follow-up. In conclusion, self-sampling increased attendance rates and was feasible and well received. This study lends further support to the proposal that self-sampling may be a valuable alternative for increasing cervical cancer screening coverage in Norway.

## Introduction

Significant reductions in cervical cancer mortality have been observed in European countries with organized and effective cytology-based cervical cancer screening programs [1]. As of today, the vast majority of cervical cancer screening programs rely still on cytology as the primary screening modality, yet screening based on high risk human papillomavirus (hrHPV) testing has been shown to provide greater protection against cervical cancer than cytology in randomized controlled trials [2]. Based on the strength of evidence, hrHPV-based cervical cancer screening has been implemented in selected regions of Norway for women 34–69 years of age in 2015 [3].

The reductions in cervical cancer incidence and mortality that can be achieved by organized screening programs depend upon the implementation of the program; high attendance and coverage are needed in order to successfully detect and treat cervical cancer in pre-invasive or early stages [4–6]. In Norway with a call-recall system, 45% of women with stage I and 10% of women with stage IV cancers had adequate screening history [7]. Women cited anticipation of pain, forgetting to make an appointment/no time, or embarrassment as reasons for non-attendance to cervical cancer screening [8–10], as well as lack of awareness of the recommended screening interval [11,12].

One of the advantages of HPV testing is the possibility for women to perform self-sampling. Indeed, self-sampling has proven to be a viable alternative to samples taken by health care professionals, and holds promise to raise screening attendance among women who do not regularly attend screening [13]. In 2008–2012 the overall 5-year coverage of the Norwegian Cervical Cancer Screening Programme (NCCSP) was 74.9% (25–69 years of age), but among women 26–29 years of age coverage was only 60%. A small increase in cervical cancer incidence over the last 10 years in women under 35 years of age in Norway [14] underlines the need for counter measures.

The purpose of the SElf-SAMpling (SESAM) study was to demonstrate the effect of self-sampling among women who do not attend the NCCSP. It is the first time self-sampling has been studied in the NCCSP. In particular, we assessed 1) impact of the self-sampling on screening attendance and coverage; 2) the performance of two different self-sampling devices for hrHPV testing; 3) women’s experience of the two self-sampling devices used, as well as reasons for not otherwise attending the NCCSP.

## Material and Methods

### Setting

Until 2015, the NCCSP recommended that women aged 25–69 years undergo cytology-based screening every 3 years. An information letter describing the purpose of the NCCSP is mailed to every women residing in Norway the year they turn 25 years of age. The NCCSP coordinates several registers in Norway, among them the Cytology Register, the Histology Register, the HPV Register, and the Cervical Intraepithelial Neoplasia treatment (CIN) Register. All cervical cytology results in Norway obtained by conventional cytology and liquid-based cytology (LBC), regardless of whether they were obtained in a public or private setting, have been recorded in the Cytology Register since 1991, all histological diagnoses from cervical biopsies or treatment specimens have been registered in the Histology Register since 2002, and all HPV test results have been registered in the HPV Register since 2005. The CIN Register contains records of treatments of cervical precancerous lesions since 1997. The reporting is mandatory and ensures a close to 100% completeness of records in the registries. Using the personal identification number, an 11-digit number that is unique for each resident in Norway, NCCSP administrators can perform record linkage between the screening registers and the National Population Register to capture individual screening histories. NCCSP administrators use this information to contact women with inadequate screening histories in the form of a reminder letter. Shortly before 3 years have passed since a woman’s last recorded test result, the NCCSP sends a 1^st^ reminder. If there is no cytology result recorded in the 12 months following the 1^st^ reminder, a 2^nd^ reminder is mailed. Each woman is responsible for scheduling her own screening appointment. A complete description of the NCCSP has been published elsewhere [15].

### Study population, randomization and allocation

Our study population was comprised of non-attenders to the NCCSP. We defined a non-attender as a woman 26–69 years of age without any cytology, HPV, or histology result recorded in the NCCSP registries within 12 months of the 1^st^ reminder, i.e. those who were due to receive the 2^nd^ reminder. 3393 non-attenders were identified in Oslo in April/May 2013. We identified by electronic randomization 800 of them (300 each from the age groups 26–34 and 35–49 years, and 200 women from the age group 50–69 years) and allocated them to the *intervention group*. The remaining 2593 non-attenders served as the *control group* and were followed according to the established procedures of the NCCSP.

**Fig 1:**
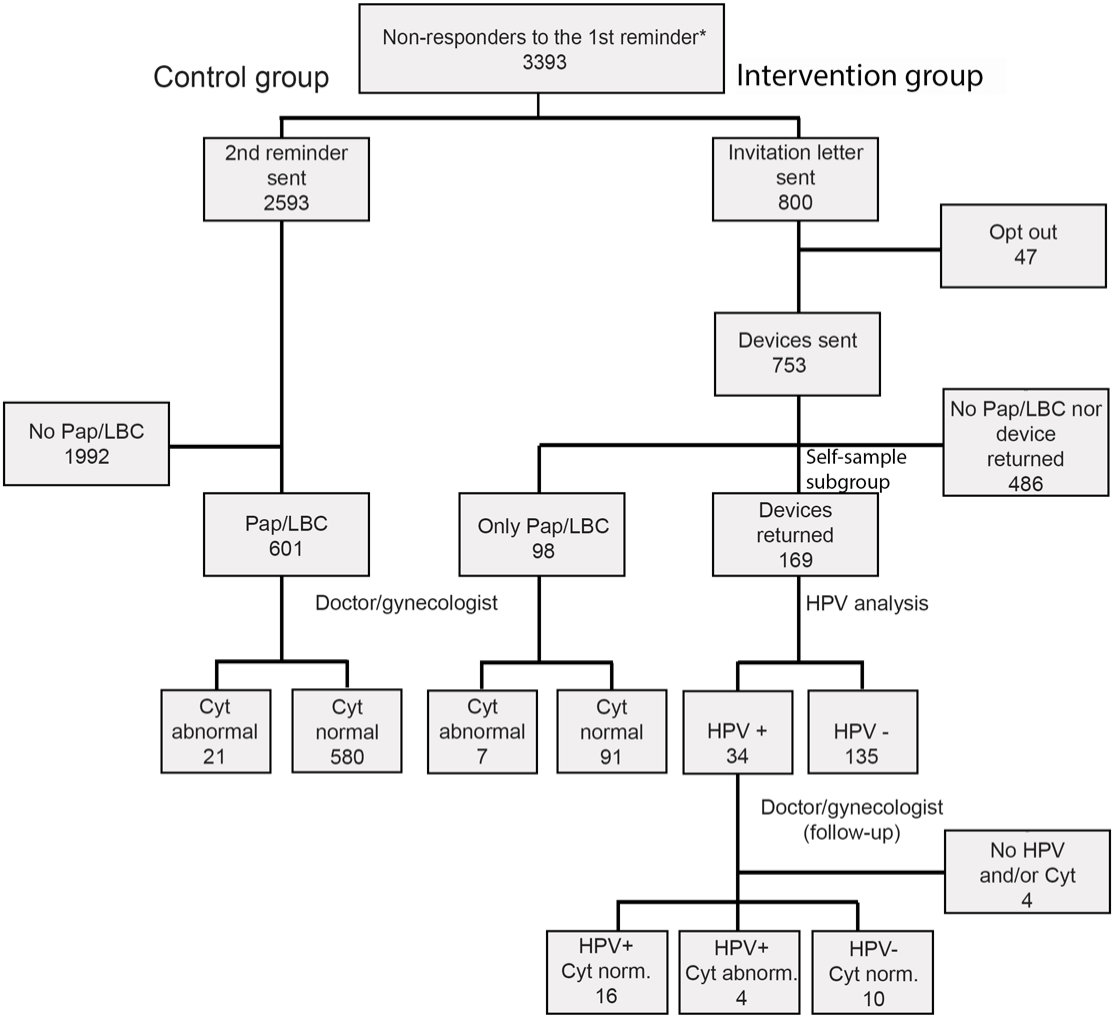
Flowchart of the study population. *Non-attender is a woman 26–69 years of age without a responder to the 1^st^ reminder measured as no cytology, HPV or histology results recorded in the NCCSP registries within 12 months of the 1^st^ reminder. HPV: human papillomavirus, Cyt: cytology.

In May 2013 we sent the 800 non-attenders an information letter, inviting them to participate in the SESAM study as part of the *intervention group*. Forty-seven women declined to participate and twenty-four were not reached; their information letters were returned due to wrong addresses. About 3 weeks later, after electronic randomization one of two self-sampling devices were randomly mailed to each of the 729 women along with user instructions, an informed consent form, a pre-paid return envelope, and a questionnaire to collect information on attitudes towards and ease of self-sampling, and to identify reasons why women did not attend the NCCSP according to recommendations. We received 177 completed questionnaires and signed informed consent forms from women in the intervention group. One hundred sixty-nine women returned the self-sampling device (self-sampling subgroup), whereas 98 women in the intervention group visited a physician for a cervical smear (Fig 1).

### Ethical statement

The study was approved by South Eastern Norway Regional Ethical Committee, 2013/402/REK South-East B. From the self-sampling group, signed informed consents was obtained from all participants included in the study, while from the control group exemption from signed informed consent was given by the Regional Ethical Committee.

### HPV testing in self-sampling devices

Two self-sampling devices were used in this study: the Delphi Screener^TM^, a lavage-based sampler (Delphi Bioscience, Scherpenzeel, the Netherlands) and the Evalyn Brush, which is a dry brush (Rovers Medical Devices B.V., Oss, the Netherlands).

All self-sampled specimens were sent for hrHPV testing at the Molecular Pathology Laboratory, Copenhagen University Hospital, Hvidovre, Denmark. All samples were processed into two aliquots; one to be analyzed using CLART® HPV2 test (Genomica, Madrid, Spain), the other to be analyzed using *digene*® Hybrid Capture 2 (HC2) test (QIAGEN, Hilden, Germany). CLART is a genotyping assay detecting 13 individual hrHPV types and 22 low-risk HPV types. HC2 detects 13 hrHPV types as a bulk “yes/no” result without genotyping. The 13 types here called high risk (hr) are the 12 defined by the International Agency for Research on Cancer as carcinogenic to humans (Group 1:HPV16, 18, 31, 33, 35, 39, 45, 51, 52, 56, 58, 59) and one defined as probably carcinogenic to humans (Group 2A: HPV68) [16].

The return volume of the Delphi Screener was 0.1–4.0 ml. When the return volume was below 3 ml, Qiagen Standard Transport Medium (STM) was added to achieve a final volume of 3 ml for processing, acknowledging the inherent dilution this causes. Cell pellets were collected by centrifugation for 10 min at 3000 rpm and re-suspended in 1 ml of STM, which was the starting point for HPV testing. The Evalyn Brush processing included brush tip removal into a vial with 4 ml of STM, then vortexed for 3x15 s. Following this, the brush head was removed from the vial and the sample was centrifuged for 10 min (3000 rpm), and the supernatant was discarded. The pellet was re-suspended in 1 ml STM for end-point analysis.

For testing by CLART, 0.2 ml of the re-suspended sample was spun down (5 min, 14,000 rpm), with the supernatant removed and the cell pellet re-suspended in a mix of 180 μl phosphate buffered saline (10x conc. pH 7.4, pharmacy product) and 20 μl Proteinase K (recombinant, PCR Grade, Roche Diagnostics GmbH, Mannheim, Germany). Samples were then vortexed and incubated for 1 hour at 56°C and 1 hour at 90°C. HPV DNA was purified using the MagNa Pure LC 96 instrument with the MagNA Pure LC Total Nucleic Acid Isolation Kit (all Roche Diagnostics GmbH, Mannheim, Germany). Five μl of purified DNA were used for the PCR amplification. Visualization was performed on the CLART HPV2 microarray and analyzed using the CAR® (Clinical Array Reader) unit in concordance with the manufacturer’s specifications, except that we used 10 μl of the denatured PCR products for the resulting visualization (Genomica, Madrid, Spain). All samples with an invalid outcome were retested, and the second result was considered definitive. Using this protocol 96.4% of all the self-sampled specimens yielded a valid result, with an increase to 98.2% (166/169) when invalid samples were retested as described above.

HC2 is a hybridization assay without an internal control for the sufficiency of sample material. 0.5 ml of the sample was pretreated manually (DNA denaturation) prior to testing according to the manufacturer’s protocol (Qiagen, Hilden, Germany). Testing of these samples was performed on an automated Rapid Capture^®^ System (Qiagen, Gaithersburg, Maryland, USA), using pretreatment dependent scripts supplied by Qiagen. The cut-off hrHPV-positive specimens’ was 1.0 RLU/CO according to the manufacturer’s recommendations presented as relative light unit/cutoff (RLU/CO) ratio.

### Follow-up cytology and HPV testing in the self-sampling subgroup

Women in the self-sampling subgroup were sent a letter with their HPV test result. Women who were hrHPV-positive by either CLART and/or HC2 also received a scheduled appointment with a gynecologist to have a follow-up specimen collected. Gynecologists were informed about the study and the reason for referral (hrHPV-positive in the self-sampled specimen). Follow-up specimens were collected for Liquid based cytology and HPV testing using ThinPrep® PreservCyt® Solution (Hologic Inc., Marlborough, Massachusetts). Two women preferred to visit their regular physician for cytology without HPV testing. Two women did not attend their scheduled appointment and had no cytology results in the Cytology Register. Follow-up specimens were sent for LBC testing first, after which the remainder of the specimens was then sent for HPV testing as described above. For safety purposes, one woman who was positive for HPV66 and two women with a HPV positivity signal close to the threshold for HPV45 and HPV51 also received a scheduled appointment with a gynecologist. Follow-up specimens for these two women showed normal cytology and HPV-negative results.

### Statistical analysis

Screening attendance was defined as either returning a self-sampling device and/or having a cervical smear taken by a physician (for conventional cytology or LBC) between April 2013, when non-attenders were identified, and the end of 2013. The results reflects an intention-to-treat population. Relative risks (RRs) and 95% confidence intervals (Cis) were calculated. To determine the agreement between CLART and the HC2 test in self-sampled specimens we used Kappa coefficients with 95% confidence intervals (CI). Classification of agreement was done according to Landis and Koch [17]. Calculations of HPV prevalence and questionnaire responses, as well as all statistical analyses were done in Stata 13 (StataCorp. 2013, Stata Statistical Software: Release 13, College Station, Texas, USA).

## Results

### Screening attendance

In all age groups the attendance rate was higher in the intervention group than the control group, with an overall attendance rate of 33.4% versus 23.2% (Table 1). In total, 267 women from the intervention group attended screening; 169 (63.3%) did so by returning the self-sampling device, while the remaining 98 (36.7%) made an appointment with a physician. The oldest age group had a slightly higher proportion of women returning self-sampling device among those that participated. Among the 98 participants that underwent clinical sampling in the intervention group 26 had a sample taken before they received the self-sampling device.

**Table 1:** Attendance rates in the intervention group and the control group by age and self-sampling device.

		Intervention group							Control group			Total participation HPV/control arm	
		Total			HPV self-test		Cytology		Cytology				
		Invited	Participants		Participants		Participants		Invited	Participants			
		N	n	%	n	%	n	%	N	n	%	RR	95% CI
	HPV Delphi	150	47	31,3	30	20,0	17	11,3					
26–34	HPV Evalyn	150	56	37,3	32	21,3	24	16,0					
	Total	300	103	34,3	62	20,7	41	13,7	848	184	21,7	1,58	1.29–1.94
	HPV Delphi	150	49	32,7	27	18,0	22	14,7					
35–49	HPV Evalyn	150	45	30,0	31	20,7	14	9,3					
	Total	300	94	31,3	58	19,3	36	12,0	981	240	24,5	1,28	1.05–1.56
	HPV Delphi	100	38	38,0	24	24,0	14	14,0					
50–69	HPV Evalyn	100	32	32,0	25	25,0	7	7,0					
	Total	200	70	35,0	49	24,5	21	10,5	764	177	23,2	1,51	1.20–1.90
	HPV Delphi	400	134	33,5	81	20,3	53	13,3					
Total	HPV Evalyn	400	133	33,3	88	22,0	45	11,3					
	Total	800	267	33,4	169	21,1	98	12,3	2593	601	23,2	1,44	1.28–1.62

N: Number of enrolled women, n: number of attenders in the category

### HPV positivity rates

The overall hrHPV prevalence (by HC2 or CLART) in the self-sampling subgroup was 20.1% (34/169 samples, Table 2). HPV prevalence was 29% at age 26–34 years, 17% at age 35–49 years, and 12% at age 50–69 years. Age group specific and overall HPV prevalence was similar independent of self-sampling device.

**Table 2:** High-risk human papillomavirus (hrHPV) positivity by age, HPV test, and self-sampling device.

			HPV test					
			HC2 or CLART		HC2		CLART	
Age (years)	Self sampling device	N	Positive	%	Positive	%	Positive	%
**26–34**	**Delphi Screener**	30	8	26.7	5	16.7	8	26.7
	**Evalyn Brush**	32	10	31.3	8	25.0	8	25.0
	** **	**62**	**18**	**29.0**	**13**	**21.0**	**16**	**25.8**
**35–49**	**Delphi Screener**	27	4	14.8	4	14.8	1	3.7
	**Evalyn Brush**	31	6	19.4	5	16.1	4	12.9
	** **	**58**	**10**	**17.2**	**9**	**15.5**	**5**	**8.6**
**50–69**	**Delphi Screener**	24	4	16.7	3	12.5	2	8.3
	**Evalyn Brush**	25	2	8.0	2	8.0	1	4.0
		**49**	**6**	**12.2**	**5**	**10.2**	**3**	**6.1**
**Total**	**Delphi Screener**	81	16	19.8	12	14.8	11	13.6
	**Evalyn Brush**	88	18	20.5	15	17.0	13	14.8
	** **	**169**	**34**	**20.1**	**27**	**16.0**	**24**	**14.2**

Positive = number of hrHPV positive samples based on 13 hrHPV types detectable by both devices (16,18,31,33,35,39,45,51,52,56,58,59,68). HC2: hybrid capture 2. N = Number of women returning self-sampling device.

The overall agreement for hrHPV positivity between CLART and HC2 was 89.9% (95% CI: 84.4%-94.0, kappa 0.61, 95% CI: 0.44–0.78) (Table 3). Agreement between CLART and HC2 for the Delphi Screener was 0.54 (95% CI: 0.28–0.81) and for the Evalyn Brush was 0.66 (95% CI: 0.44–0.88). Among the ten CLART-negative and HC2-positive for hrHPV, three of the CLART-hrHPV-negative were positive for low-risk HPV types (HPV53, 66 and 70, 83) (S1 Table). Compared to all samples that were HC2-positive for hrHPV, the discordant samples positive with HC2 had lower average signal strength (mean: 12.4 versus 20.5 RLU/CO, median: 3.33 versus 4.85 RLU/CO). The Delphi screener had a variable volume upon return, with a mean of 1.9 ml (range: 0.1–4.0 ml); however there was no observed relation between volume and hrHPV positivity rates (S2 Table).

**Table 3:** Concordance/discordance between CLART and Hybrid Capture 2 (HC2).

		CLART		
		hrHPV-positive	hrHPV-negative	Total
**HC2**	**hrHPV-positive**	17 (10.1%)	10 (5.9%)	27 (16.0%)
	**hrHPV-negative**	7 (4.1%)	135 (79.9%)	142 (84.0%)
	**Total**	24 (14.2%)	145 (85.8%)	169 (100%)

HC2: hybrid capture 2. hrHPV: high-risk human papillomavirus based on 13 hrHPV types detectable by both devices (16,18,31,33,35,39,45,51,52,56,58,59,68). For all HPV types detected with CLART see S1 Table.

### HPV types

**Fig 2:**
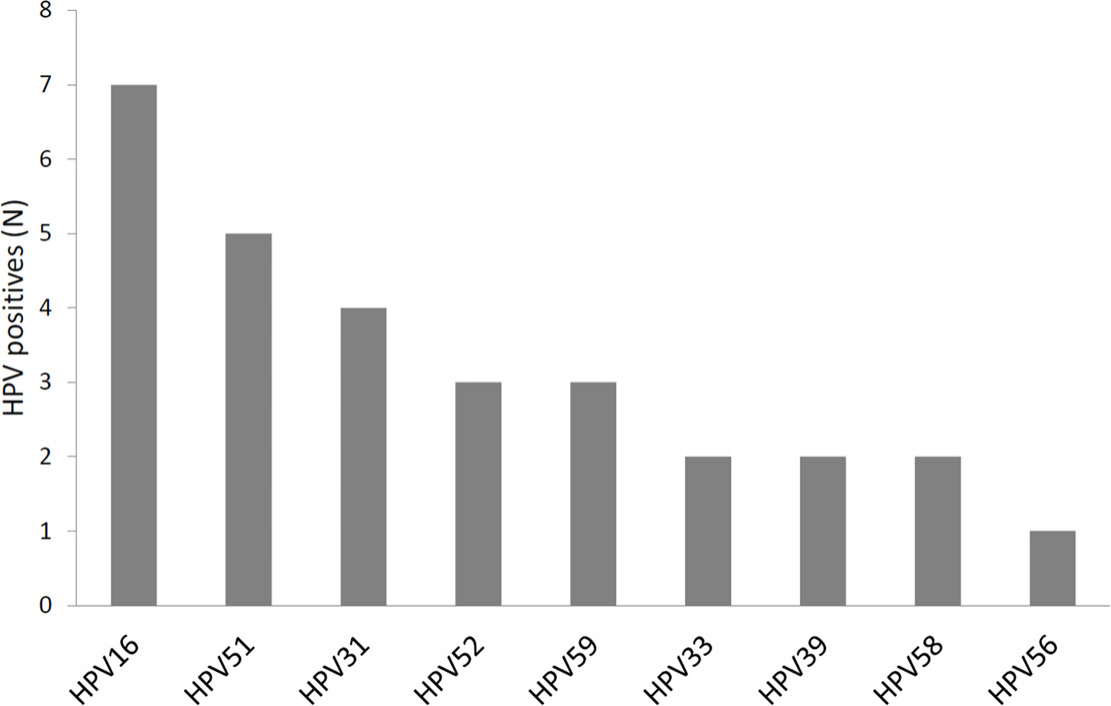
hrHPV type distribution of 34 hrHPV-positive samples by CLART, including single and multiple hrHPV infections. hrHPV: high-risk human papillomavirus, based on 13 hrHPV types detectable by both devices (16,18,31,33,35,39,45,51,52,56,58,59,68).

HPV16 was the most common HPV type identified, followed by HPV51 and HPV31 (Fig 2). Among the 13 hrHPV types, nine were detected at least once. Notably, HPV18 was not identified at all. Fourteen out of 24 (58.3%) hrHPV-positive women had multiple HPV infections, of which three were infected with at least two hrHPV type S1 Table.

### Follow-up of women

In the control group, 3.5% (21/601) had abnormal cytology results that required follow-up, in addition to 3.3% that had an unsatisfactory cervical smear. In the intervention group 7.1% (7/98) of women undergoing clinical sampling and 20.1% (34/169) of women performing self-sampling had a result that required follow up. Out of 34 women with hrHPV-positive results by either CLART or HC2, 30 attended a scheduled appointment with the gynecologist and two chose instead to see their physician for a follow-up specimen. Four of these 34 women had abnormal cytology result in their follow-up specimen (S3 Table). In addition, the follow-up specimens of the 30 women who attended their scheduled appointment were tested for HPV by CLART and HC2; 13 (43.3%) were hrHPV-positive by CLART (S1 Table). In 11 of these 13 samples at least one of the hrHPV types found at follow-up was also found in self-sampled specimen. All women were further followed up as necessary according to the guidelines of the NCCSP.

### Self-sampling experience and reasons for non-attendance

The majority of women in the self-sampling subgroup found the self-sampling procedure to be easy and not painful, embarrassing, or scary (Table 4). Seventy-seven percent of women either agreed or partly agreed that they performed the self-sampling successfully. The Delphi Screener and the Evalyn Brush received similar responses for all questions, but there was a trend towards a higher percentage of women who “agreed” than “partly agreed” that taking the test was easy and that it was performed successfully, and they were less unsure during the sampling procedure when the Evalyn Brush was used. The main reasons cited for not attending the NCCSP were 1) not remembering having received the 1^st^ reminder (39%), 2) forgetting to make an appointment (33%)3) did not like physician taken sample procedure (22%), 4) lack of time (14%) (S4 Table).

**Table 4:** Self-sampling experience taken from the questionnaire.

		Agree		Partly agree		Do not agree		No opinion	
Questions	N	n	%	n	%	n	%	n	%
I believe taking the test was easy									
Delphi Screener	78	64	82	14	18	0	0	0	0
Evalyn Brush	88	82	93	6	7	0	0	0	0
Total	166	146	88	20	12	0	0	0	0
I believe I performed the test successfully									
Delphi Screener	71	51	72	19	27	1	1	0	0
Evalyn Brush	71	58	82	9	13	2	3	2	3
Total	142	109	77	28	20	3	2	2	1
I felt unsure during the sampling procedure									
Delphi Screener	72	7	10	22	31	38	53	5	7
Evalyn Brush	71	6	8	15	21	49	69	1	1
Total	143	13	9	37	26	87	61	6	4
I felt taking the test was uncomfortable									
Delphi Screener	70	1	1	7	10	62	89	0	0
Evalyn Brush	68	1	1	9	13	57	84	2	3
Total	139	2	1	16	12	119	86	2	1
I felt taking the test was painful									
Delphi Screener	70	0	0	1	1	68	97	1	1
Evalyn Brush	70	1	1	3	4	64	91	2	3
Total	140	1	1	4	3	132	94	3	2
I felt taking the test was embarrassing									
Delphi Screener	70	1	1	3	4	63	90	3	4
Evalyn Brush	69	1	1	3	4	63	91	2	3
Total	139	2	1	6	4	126	91	5	4
I believe taking the test was scary/filled me with anxiety									
Delphi Screener	70	0	0	3	4	65	93	2	3
Evalyn Brush	70	1	1	2	3	64	91	3	4
Total	140	1	1	5	4	129	92	5	4

N: total answers, n: number of answer in the category.

## Discussion

### Main findings

Attendance to cervical cancer screening increased from 23.2% to 33.4% when non-attenders had the choice between home-based self-sampling and making appointment to have a sample collected by a physician, compared to the control group that only had the latter option. Providing the option of self-sampling to all non-responders has the potential to increase the 5-year coverage of the NCCSP. Importantly, all 169 devices returned contained sufficient biological material for HPV testing. Among the 34 hrHPV-positive women, 32 attended a follow-up visit, which may indicate that a HPV positive result to a self-sample acts as a motivator for completing follow-up. We received no significant differences in user feedback regarding the two self-sampling devices.

Offering self-sampling to non-attenders has been suggested as a method to recruit women who do not regularly attend screening programs [18]. To increase program coverage, the NCCSP mails reminders to women who have not been screened in the last 3–4 years, encouraging them to contact their physician for cervical cancer screening. In 2012, 145 000 2^nd^ reminders were mailed. The 6-month response rate to these letters was 18.8% in the 25-69-year age group, 16.1% in the 25-29-year age group, and 18.3% in the 30-34-year age group, showing that a 2^nd^ reminder is slightly less effective in stimulating younger women to attend screening. Offering the possibility of self-sampling increased attendance also in the youngest age group in our study, which suggests that it is a suitable strategy across different age groups. However, it should be noted that overall, 37% of the women in the intervention group who attended screening preferred to visit a physician for clinical sampling, suggesting that self-sampling should serve as a co-strategy, and should not be offered as the only option.

This SESAM study increased cervical screening attendance among non-attenders from 23% to >33% by offering self-sampling. This increase is similar to that observed in other studies that targeted non-attenders in invitation-based cervical cancer screening programs [13]. In the present study, no difference in attendance rate could be attributed to the random allocation of the Delphi Screener and the Evalyn Brush. In general, women answered the questionnaire favorably on both devices. Finally, not remembering receiving a call for screening was listed as the most dominant cause of non-attendance.

In 2011 in the NCCSP, 27 416 women responded to the 145 725 second reminder letter, which gives an attendance rate of 18.8% [19]. The attendance rate of the corresponding control group in this study was 23.2%, which may partly be because we allowed for a follow-up time of about 8 months in the control group. Increasing the response rate to the second reminder from 23.2% to 33.4% may result in ~11 000 more women attending the NCCSP yearly. Self-sampling would address several of the reasons our study women gave for not attending regular screening, including “I forgot to make an appointment”, “I do not like that my general practitioner performs the sampling” and “I do not have the time”. However, the most common reason cited, not remembering receiving a reminder letter, might not be directly addressed by offering self-sampling.

From a program point of view, a higher proportion of women in the self-sampling subgroup were hrHPV-positive, and thus they required more intensive follow-up compared to women with abnormalities that requires follow-up in the control group. This fits well with previous studies comparing the performance of hrHPV and cytology as screening tests. It is well recognized that the clinical sensitivity of hrHPV testing is higher than that of cytology, leading to a lower risk of cervical cancer after a negative test [2]. However, the lower specificity of HPV testing means that more women are referred to follow-up, which may lead to a higher rate of colposcopies and treatment procedures [20]. Here, we showed that 10% (13+4/169) of the women returning self-sampling specimens were hrHPV-positive by both self-sampling and the later, clinician collected, follow-up sample. In comparison, 3.5% (21/601) of the control group had abnormal cytology results that required follow-up, in addition to 3.3% that had an unsatisfactory cervical smear. Our study suggests that about 1500–2200 (14–20% of 11 000) women will test hrHPV-positive annually if self-sampling becomes an option for non-attenders, implying that, if implemented, extra resources should be (re)allocated. Given the high rate of cervical cancer among non-attenders, a careful evaluation of clinically relevant end-points is needed to define the best possible screening methods for non-attenders.

### Methodology and HPV results

We used two HPV tests: CLART and HC2. Though the numbers from this study are very small, overall the two assays showed substantial agreement, regardless of the self-sampling device. However, with only 34 hrHPV-positive samples, this study does not have the power to detect any differences in test performance by age or self-sampling device, the latter in particular since each woman only used one of the self-sampling devices. The general decrease in hrHPV positivity with increasing age is in line with other studies [21] and a comparable percentage of women in the Delphi and the Evalyn group were hrHPV-positive.

The most prevalent HPV type found was HPV16 [21]. Although only 24 samples were positive by the CLART assay, which could identify individual genotypes, we found nine out of the 12 hrHPV types identified by the International Agency for Research on Cancer as carcinogenic to humans. We observed some discrepancies when comparing HPV types identified by CLART and HC2. This has recently been more thoroughly analyzed in 5064 samples obtained by physicians from women attending routine screening in Denmark [22]. Here, the agreement between CLART and HC2 for HPV detection was 50%, which is similar to the assay agreement presented here, although our numbers are smaller. Rebolj et al [12] has suggested that assay design is the most likely reason for the discrepancies between these tests, and it has previously been shown that some discrepancy between HC2 and other HPV assays may be due to known cross-reactivity with HPV66 and HPV70 in HC2 [23–25].

Overall, processing self sample specimens was easy on CLART who relies on third party DNA extraction, in our case Roche MagNaPure, and once the DNA has been extracted multiple tests can be performed on the same extract. The internal human CFTR gene control on CLART furthermore means that negative HPV tests can be validated for sufficient sample which is a crucial quality control element for self taken samples, where the quality presumably can vary. However, the HC2 was more laborious and sample consuming, not allowing for repeat testing on the same sample, and the lack of an internal control for sufficiency on HC2 means that the assay can not be quality controlled to the same extend for sample sufficiency compared to PCR based assays like CLART, in theory allowing for false negative test results.

### Strengths and weaknesses of the study

A weakness of this SESAM study is the restriction to an urban region (Oslo) where the 3.5-year attendance rate is 2.9% lower than the national average. Moreover, women’s attitude towards self-sampling in this area may be different than in other regions of Norway. It took approximately two months from the identification of non-attenders until they received the self-sampling device. ~1/4 of women in the self-sampling group underwent clinical sampling during this period, but they may have opted for a self-sample test if it had been received earlier. Our information letter was written in Norwegian only, while the reminder letters in the NCCSP contain the address of a website where information can be obtained in English. As many immigrants in Norway are from Western countries, in the future instructions and information may be provided in English or other languages to further increase response rates. The main strength of the study is the minor loss in the follow-up of hrHPV-positive women and that we were able to confidently measure this loss through the Cytology Registry using each woman’s personal identification number.

### Perspectives

The optimal self-sampling strategy for increasing attendance will need to be balanced with the costs of self-sampling. In this study, we gave women the opportunity to opt out of the study. A recent meta-analysis showed that this strategy can increase participation in both the per-protocol and intention-to-treat groups [26]. The latter additionally includes data on women invited to perform self-sample but instead chose to have a Pap smear taken by a clinician [26] and is similar to the analysis presented here. The alternative strategy is an opt-in strategy, in which women actively need show their interest (e.g. sign up or collect device at a given location) in using self-sampling based on instructions in an information letter. Although in general not able to increase participation in Italy [9,27], an intention-to-treat analysis based on a study performed in Sweden showed significant increase in participation [28]. An opt-in strategy may lower costs as fewer devices are distributed, thereby reducing device procurement and postage costs, but more knowledge about how to optimize such a strategy is needed. Future studies should also investigate the attendance in subsequent screening rounds among women preferring self-sampling. An option could be to send a self-sampling device along with the 1^st^ reminder to such women.

## Conclusion

In conclusion, this study shows that offering self-sampling to non-attenders in the NCCSP will increase the overall attendance rate. Both the Delphi Screener and the Evalyn brush were positively received by the women using them, and collected satisfactory samples for HPV testing by CLART and HC2. Overall, this study underlines the potential of self-sampling among non-attenders in the NCCSP as a feasible method for increasing attendance rates.

## Supporting Information

S1 Table Genotype distribution and cytology diagnosis for all high-risk human papillomavirus (hrHPV)-positive women from self-sampled specimens and follow-up specimens taken by physicians. (DOCX)

S2 Table Delphi screener return volumes. (XLSX)

S3 Table Follow-up cytology of high-risk human papillomavirus (hrHPV)-positive women in the self-sampling subgroup. (XLSX)

S4 Table Questionnaire: Reasons for not attending the national screening program. (XLSX)
