# Supplementary material for: Self-Sampling for Human Papillomavirus Testing among Non-Attenders Increases Attendance to the Norwegian Cervical Cancer Screening Programme
Source: PLoS One. 2016 Apr 13;11(4):e0151978. doi: 10.1371/journal.pone.0151978 (PMC4830596; doi:10.1371/journal.pone.0151978)
Supplement: S1 Table — (DOCX) [file pone.0151978.s001.docx]

Table S1. Genotype distribution and cytology diagnosis for all high-risk human papillomavirus (hrHPV)-positive women from self-sampled specimens and follow-up specimens taken by physicians.

|  | **Self-sampling** | | | | | **Follow-up** | | | | |
| --- | --- | --- | --- | --- | --- | --- | --- | --- | --- | --- |
|  | HC2 hrHPV | CLART hrHPV | CLART HPV type | HC2: Relative Lights Unit/Cut off (Positive≥1.0) | Return volume (ml) | Cytology diagnosis | HC2 hrHPV | CLART hrHPV | CLART HPV type | HC2: Relative Lights Unit/Cut off (Positive≥1.0) |
| **Delphi** | + | + | 31,33,52 | 71.37 | 1.5 | LSIL | + | + | 31,33,52 | 76.49 |
|  | + | + | 51,83 | 2.41 | 1.5 | Normal | + | + | 51 | 1.02 |
|  | + | + | 16 | 4.85 | 3.0 | Normal | + | + | 16 | 10.76 |
|  | + | + | 56,39 | 195.27 | 2.0 | Normal | + | + | 56 | 1707.90 |
|  | + | + | 31,83 (61) | 1.40 | 1.0 | Normal | . | . | . | . |
|  | + | + | 33 | 3.80 | 3.0 | Normal | - | + | 33 | 6.93 |
|  | + | + | 59 | 21.12 | 1.0 | Normal | - | - | Negative | 0.18 |
|  | + | - | 53,66 | 2.56 | 3.5 | Normal | - | - | 66 | 0.17 |
|  | + | - | 70,83 | 40.49 | 1.5 | Normal | - | - | 70 | 0.30 |
|  | + | - | Negative | 7.34 | 1.5 | Normal | - | - | Negative | 0.21 |
|  | + | - | Negative | 1.53 | 0.5 | Normal | - | - | Negative | 0.30 |
|  | + | - | Negative | 1.64 | 2.0 | Normal | - | - | Negative | 0.13 |
|  | - | + | 51,54 | 0.19 | 1.0 | Normal | + | + | 51 | 11.56 |
|  | - | + | 51 | 0.37 | 1.0 | Normal | . | . | . | . |
|  | - | + | 58,83 | 0.58 | 2.0 | Normal | - | - | Negative | 0.17 |
|  | - | + | 58,83 | 0.18 | 1.0 | Normal | - | - | Negative | 0.21 |
| **Evalyn** |  |  |  |  |  |  |  |  |  |  |
|  | + | + | 31,42 | 8.65 | . | Normal | + | + | 39 | 16.29 |
|  | + | + | 16 | 13.00 | . | Normal | + | + | 16 | 1.45 |
|  | + | + | 16,66 | 3.00 | . | Normal | + | - | 66 | 90.49 |
|  | + | + | 16 | 18.45 | . | Normal | + | + | 16 | 2.20 |
|  | + | + | 16,52,53,70 | 5.87 | . | ASC-H | + | + | 16 | 91.37 |
|  | + | + | 51,7 | 1.63 | . | ASC_US | + | + | 51,82 | 16.93 |
|  | + | + | 16 | 1.23 | . | HSIL | + | - | No DNA | 5.27 |
|  | + | + | 39, (89) | 11.47 | . | Normal | - | - | Negative | 0.18 |
|  | + | + | 52,61 | 49.33 | . | Normal | - | - | Negative | 0.17 |
|  | + | + | 51,62 | 16.27 | . | Normal | - | - | 62 | 0.16 |
|  | + | - | Negative | 2.12 | . | Normal | + | - | 66 | 1.70 |
|  | + | - | 70,83 | 57.29 | . | Normal | + | - | 70,83 | 106.08 |
|  | + | - | Inhib | 1.92 | . | Normal | - | + | 59 | 0.28 |
|  | + | - | Negative | 4.10 | . | Normal | - | - | Negative | 0.16 |
|  | + | - | Negative | 4.53 | . | . | . | . | . | . |
|  | - | + | 16 | 0.15 | . | Normal | + | + | 16 | 6.17 |
|  | - | + | 59,61 | 0.32 | . | . | . | . | . | . |
|  | - | + | 31 | 0.26 | . | Normal | - | - | Negative | 0.19 |

hrHPV positive by CLART is defined as: 16,18,31,33,35,39,45,51,52,56,58,59,68 (13 types, bold).

CLART types in parenthesis is below threshold. HC2: Hybrid Capture 2.
